# Lying Awake at Night: Cardiac Autonomic Activity in Relation to Sleep Onset and Maintenance

**DOI:** 10.3389/fnins.2019.01405

**Published:** 2020-01-15

**Authors:** Marina Nano, Pedro Fonseca, Sebastiaan Overeem, Rik Vullings, Ronald M. Aarts

**Affiliations:** ^1^Department of Electrical Engineering, Eindhoven University of Technology, Eindhoven, Netherlands; ^2^Philips Research, Eindhoven, Netherlands; ^3^Sleep Medicine Centre Kempenhaeghe, Heeze, Netherlands

**Keywords:** sleep, ECG, heart rate variability, HRV, sleep onset latency, wake after sleep onset, sleep onset, sleep maintenance

## Abstract

Insomnia, i.e., difficulties initiating and/or maintaining sleep, is one of the most common sleep disorders. To study underlying mechanisms for insomnia, we studied autonomic activity changes around sleep onset in participants without clinical insomnia but with varying problems with initiating or maintaining sleep quantified as increased sleep onset latency (SOL) and wake after sleep onset (WASO), respectively. Polysomnography and electrocardiography were simultaneously recorded in 176 participants during a single night. Cardiac autonomic activity was assessed using frequency domain analysis of RR intervals and results show that the normalized spectral power in the low frequency band (*LF*_*nu*_) after sleep onset was significantly higher in participants with long SOL compared to participants with short SOL. Furthermore, the normalized spectral power in the high frequency band (*HF*_*nu*_) was significantly lower in participants with long SOL as compared to participants with short SOL over 3 time periods (first 10 min in bed intending to sleep, 10 min before, and 10 min after sleep onset). These results suggest that participants with long SOL are more aroused in all three examined time periods when compared to participants with short SOL, especially for young adults (20–40 years). As there is no clear consensus on the cutoff for an increased WASO, we used a data-driven approach to explore different cutoffs to define short WASO and long WASO groups. *LF*_*nu*_, *HF*_*nu*_, and *LF*/*HF* differed between the long and the short WASO groups. A higher *LF*_*nu*_ and *LF*/*HF* and a lower *HF*_*nu*_ was observed in participants with long WASO for most cutoffs. The highest effect size was found using the cutoff of 66 min. Our findings suggest that autonomic cardiac activity has predictive value with respect to sleep characteristics pertaining to sleep onset and maintenance.

## 1. Introduction

Sleep has an important role in promoting health and many studies support a function of sleep in memory and cognitive processing. Even though sleep quality is often employed as an outcome criterion for therapeutic success (Kucharczyk et al., 2012; Carroll et al., 2015), the exact definition and quantification of sleep quality is still ambiguous. Sleep problems, in particular difficulties initiating and/or maintaining sleep are becoming more prevalent (Ohayon, 1997) and one third of the population worldwide is affected occasionally by insomnia symptoms (Ohayon, 1997; Nano et al., 2017). A clinical diagnosis of insomnia disorder requires presence of difficulties initiating and/or maintaining sleep with subsequent daytime impairments at least three nights per week and for at least 3 months. Insomnia disorder often involves medical and psychological comorbidities, making the understanding of underlying disrupted sleep mechanisms more challenging. Studying an earlier developmental stage of sleep disruption may provide new insights into these mechanisms.

Research over the past decade linked insomnia to an increased risk of hypertension, stroke, obesity, and depression (Colten and Altevogt, 2006). Several factors can be associated with problems initiating and/or maintaining sleep such as elevated levels of stress and physiological hyperarousal (Bonnet and Arand, 2010). Difficulties in sleep onset and/or maintenance can be measured by polysomnographic (PSG) recording which involves simultaneous recording and interpretation of electroencephalography, electrooculography, and electromyography. However, PSG recording is expensive, labor-intensive and inadequate for the long-term monitoring of sleep. Especially for people with insomnia, the plethora of electrodes that are attached to the body during PSG increase obtrusiveness and have a negative impact on the participants' ability to fall or stay sleep (Nano et al., 2018).

Cardiac autonomic activity is closely linked to sleep and circadian physiology, as it has been shown by the disrupted autonomic control that accompanies sleep loss (Lanfranchi and Somers, 2011; Nano et al., 2017). Heart rate variability (HRV) can be described as a set of objective measures that give information about the dynamics of the cardiac autonomic nervous system (ANS) and it has been widely used in sleep research (Chouchou and Desseilles, 2014; Shaffer and Ginsberg, 2017; Herzig et al., 2018). Several studies have demonstrated that the transition from wakefulness to sleep and vice versa are associated with changes in autonomic activity (Burgess et al., 1999a,b; Carrington et al., 2005; Shinar et al., 2006; Okamoto-Mizuno et al., 2008). For instance, various studies have shown that heart rate (HR) and blood pressure decreased during the transition from wakefulness to sleep (Burgess et al., 1999b; Carrington et al., 2003, 2005). Most research has focused on 1-h averages of HRV features between different sleep stages and wakefulness (Burgess et al., 1999b; Carrington et al., 2003; Okamoto-Mizuno et al., 2008) as well as between normal and pathological sleep (Shinar et al., 2006; Tobaldini et al., 2013). We expect that autonomic changes, especially those describing the transition from wakefulness to sleep, occur in a shorter time scale such as 10 min, as described in a recent study by Miller et al. (2016).

The aim of this work is to study difficulties in initiating and/or maintaining sleep in an early developmental stage of sleep disruption. Specifically, we aim to assess autonomic activity changes through HRV during the process of falling asleep, comparing three subsequent time periods: during the first 10 min in bed intending to sleep, during the 10 min before sleep onset, and the first 10 min after sleep onset. We compare these autonomic changes between participants with long and short sleep onset latency (SOL). In this way we aim to investigate the intention to fall asleep and the transition from wakefulness to sleep from an autonomic cardiac perspective. Furthermore, our goal is to extend the study of sleep difficulties by including also participants with difficulties maintaining sleep, quantified by wake after sleep onset (WASO). Therefore, the second objective of this study is to determine whether a state of arousal (if present) at the onset of sleep, relates to increased awakening during the night.

## 2. Materials and Methods

### 2.1. Data

The data used comprises four data sets collected in four different studies. After visual inspection recordings with low ECG quality were excluded. The first data set comprises a subset of the data collected from 1997 until 2000 in the SIESTA project (Klösch et al., 2001), consisting of 192 standardized PSG recordings of participants (101 female) with a mean ± standard deviation (SD) age of 51.4 ± 19.3 years. All participants had a Pittsburgh Sleep Quality Index (PSQI) (Buysse et al., 1989) score of less than 6. PSQI is a self-rated questionnaire assessing sleep quality (Buysse et al., 1989). A score of 0-7 suggests the absence of clinically relevant insomnia or other sleep disorders (Smith and Wegener, 2003). The participants were not using or had a history of using drug and/or alcohol. In addition, none of the participants was working at night, or had been diagnosed with a medical or mental disorder that could possible interfere with the aim of the study (Klösch et al., 2001; Fonseca et al., 2017; Nano et al., 2018). Two trained somnologists from different sleep centers scored the PSG recordings of all participants according to the R&K guidelines (Kales and Rechtschaffen, 1968). The scorings were revised by a third expert who took the final decision in case of disagreement (Klösch et al., 2001; Fonseca et al., 2017; Nano et al., 2018). The SIESTA study was performed in five different countries in seven different sleep laboratories and that is why the sampling frequencies of the ECG signals range from 200 to 400 Hz. This study was approved by the local ethical committee of each research group. More details regarding the study design can be found in Klösch et al. (2001).

The second data set used in this work was collected in 2014 and we used a subset consisting of 13 participants (6 female). The participants had a mean age of 52.7 ± 8.0 years, a PSQI (Buysse et al., 1989) score of less or equal than 7 and a mean body mass index (BMI) lower than 30 kg/m^2^ (for more details regarding the study design see Fonseca et al., 2017). The sampling frequency of the ECG signals in this data set is 200 Hz. The third data set was collected in 2015 and a subset consisting of 34 participants (22 female) with a mean age of 51.7 ± 6.9 years and a BMI lower than 37 kg/m^2^ was used (for details regarding the study design refer to Fonseca et al., 2017). The sampling frequency of the ECG signals in this data set is 500 Hz. The fourth data set was collected in 2009 and comprises PSG recordings of a single-night from 8 participants (7 female) with a mean age of 29.6 ± 10.9 years and a BMI index lower than 31 kg/m^2^. The ECG signals of this data set have a sampling frequency of 500 Hz. The latter three studies were reviewed and approved by the Internal Committee of Biomedical Experiments of Philips Research and were conducted in accordance with the Declaration of Helsinki. The participants provided their written informed consent to before participation.

A trained somnologist scored the PSG recordings of all participants from the latter three data sets according to the AASM guidelines (Iber et al., 2007). None of the participants had been diagnosed with a sleep or any mental disorder. Additionally, none of the participants were using any sleep, antidepressant, or cardiovascular medication.

For the purpose of this study the following inclusion criteria were used. A participant was included in the analysis if he/she experienced a sleep onset latency of at least 10 min from the moment participants were in bed intending to sleep. In addition, the analysis is restricted only to wake epochs (before sleep onset) or sleep epochs (after sleep onset), during the 10 min before or after sleep onset, respectively. This restriction is applied in order to determine if the autonomic function itself differs between groups, or if the observed autonomic differences are driven by the fact that participants belonging to one the groups wake up more during the first 10 min after sleep onset compared to the other group. Therefore, a participant was included if at least five consecutive 30-s epochs were annotated as awake before sleep onset and as sleep after sleep onset. Furthermore, in order to use the same night for all participants we performed our analysis on the first night of PSG recording. After applying these inclusion criteria, the resulting data set comprises 176 (98 female) participants with a mean age of 51.0 ± 17.5 years and a mean BMI index of 24.8 ± 3.7 kg/m^2^.

### 2.2. Sleep Onset Definition

Sleep onset was defined according to the R&K criteria (Kales and Rechtschaffen, 1968) as the first epoch of three consecutive 30-s epochs scored as non-awake, usually N1 sleep stage or any other non-REM sleep stage (Kales and Rechtschaffen, 1968). The R&K definition (Kales and Rechtschaffen, 1968) was chosen because it requires a longer duration of sleep to be defined as onset compared to the AASM definition (Iber et al., 2007) of sleep onset, which requires only a single epoch of non-wake. A longer duration of sleep onset indicates a higher sleep stability during the transition from wakefulness to sleep state. For most participants (137, 77.8%) all three consecutive 30-s epochs were scored as N1 while for the remaining participants, one or two epochs were scored as N2.

### 2.3. Feature Extraction

A high-pass filter with a cut-off frequency of 0.8 Hz was applied on ECG modified lead II (2nd, 3rd, and 4th datasets) and modified lead I (Siesta) signals.

Then the signals were normalized with regard to mean and amplitude. R-peaks detection was performed by using the algorithm proposed by Kathirvel et al. (2011). The R-peaks were further localized using the post-processing algorithm of Fonseca et al. (2014). We excluded RR intervals longer than 2 s, shorter than 0.3 s, or shorter than 0.6 times their previous value. The resulting RR interval time series was re-sampled at a sampling rate of 4 Hz using linear interpolation. The power spectral density was estimated with a parametric auto-regressive (AR) model (Marple, 1987). Specifically we used the modified covariance method which combines minimization of the forward and backward prediction squared errors (*modcovar* and *arma2psd* functions from the Python *spectrum* package). We chose AR over non-parametric methods such as Fast Fourier transform (FFT) because FFT makes specific assumptions about periodicity that might not always be met. For defining the “optimum order” of each sliding window of the AR model the Akaike Information criterion was used, resulting to an order range of [5, 15]. In this work, four frequency domain features were extracted using a sliding window of 5 epochs centered on each 30-s epoch, guaranteeing sufficient data (2.5 min) to capture the changes in autonomic activity as recommended by (1996). A sliding window was used to provide a good compromise between the number of data points representing the examined time period and the window length to capture a reliable HRV representation. The features were: (i) the logarithm of the ratio of the spectral power in the low frequency band (LF) from 0.04 to 0.15 Hz divided by the difference between total power and the power in the very low frequency band (VLF) (0.003–0.04 Hz), (ii) the logarithm of the spectral power in the high frequency band (HF) between 0.15 to 0.4 Hz divided by the difference between total power and the power in the VLF, (iii) the ratio of *LF* and *HF* in absolute values (1996), and (iv) the frequency of the maximum power in the HF band. The four features are denoted as *LF*_*nu*_, *HF*_*nu*_, *LF*/*HF*, and *HFfreq*, respectively.

**Figure 1:**
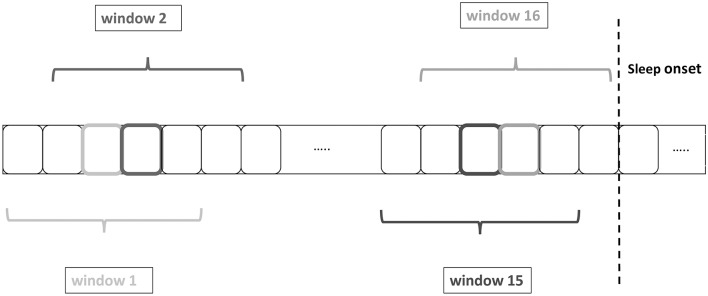
Heart rate variability (HRV) feature extraction by using a sliding windows of 2.5 min centered on each 30-s epoch. The boxes with the darker or lighter color represent the central epoch and the grayscale corresponds to the color of the window name, e.g., the 4th epoch from the left corresponds to the central epoch of window 2.

Considering that a 2.5 min sliding window is centered on every 30-s epoch, the first value of the 10 min period before sleep onset occurs at –8.75 min before sleep onset and the last value at –1.25 min before sleep onset. Accordingly, after sleep onset the first and last values of the 10 min period correspond to +1.25 and +8.75 min, yielding a total of 16 values of each HRV feature (see Figure 1). Per 10-min period, the mean value of the HRV feature values was computed.

### 2.4. Quantitative Criteria for Assessing Difficulties in Initiating and Maintaining Sleep

Lately, there have been efforts to develop standardized diagnostic criteria for the study and diagnosis of insomnia disorder (Lichstein et al., 2003; Lineberger et al., 2006). Several cutoffs for subjective SOL and WASO have been used in different studies, such as 20, 30, 40, and 60 min summarized in Lichstein et al. (2003) and Lineberger et al. (2006), with the most common being 30 min. These cutoffs have been used for investigating quantitative criteria combined with severity criteria for insomnia disorder based on subjective data. In this work, the median value of SOL in the entire data set, as measured by PSG, is 25 min, which is close to the most frequently used cutoff for SOL in the literature. Same as most literature, we used a cutoff of 30 min for dividing participants between the short and the long SOL groups. Regarding WASO, the median value in our data set was 53 min. Therefore, in addition to the most commonly used cutoff of 30 min, we investigated different thresholds to define short and long WASO groups using a data driven approach. We followed this approach for two main reasons. First, it remains uncertain which WASO cutoff is the most relevant—either in the literature or in clinical practice. Second, there is no specific physiological mechanism that would result in a specific WASO value. The different cutoffs applied in this work were determined by the 25, 40, 50, 60, and 75th percentile of the WASO of the entire data set corresponding to 28.5, 41.5, 53, 66, and 98 min, respectively, confirming a good agreement between these cutoffs and the literature summarized in Lichstein et al. (2003) and Lineberger et al. (2006).

In summary, for the study of difficulties in initiating sleep the resulting data set was divided in two groups based on the SOL according to quantitative criteria for insomnia (Lichstein et al., 2003): participants that experienced SOL greater than 30 min constitute the long SOL group, consisting of 69 participants (44 female) while the remaining comprise the short SOL group with 107 participants (54 female). The participants of both groups did not differ significantly with regard to age, BMI and sex (Mann-Whitney *U*-test and Fisher's exact test). For the study of difficulties in maintaining sleep the following divisions were considered: (i) Firstly, the data set was divided in two groups, participants with WASO greater than 30 min form the long WASO group, comprising 125 participants (65 female) while the remaining comprise the short WASO group with 51 participants (33 female). The participants of both groups did not differ significantly with regard to BMI and sex (Mann-Whitney *U*-test and Fisher's exact test). Age was significantly different between the two groups (Mann-Whitney *U*-test). (ii) Secondly, the other cutoffs that were mentioned earlier were used to divide the data set in two groups in order to investigate which cutoff gives the largest autonomic separation between the two groups.

### 2.5. Statistical Analysis

**Figure 2:**
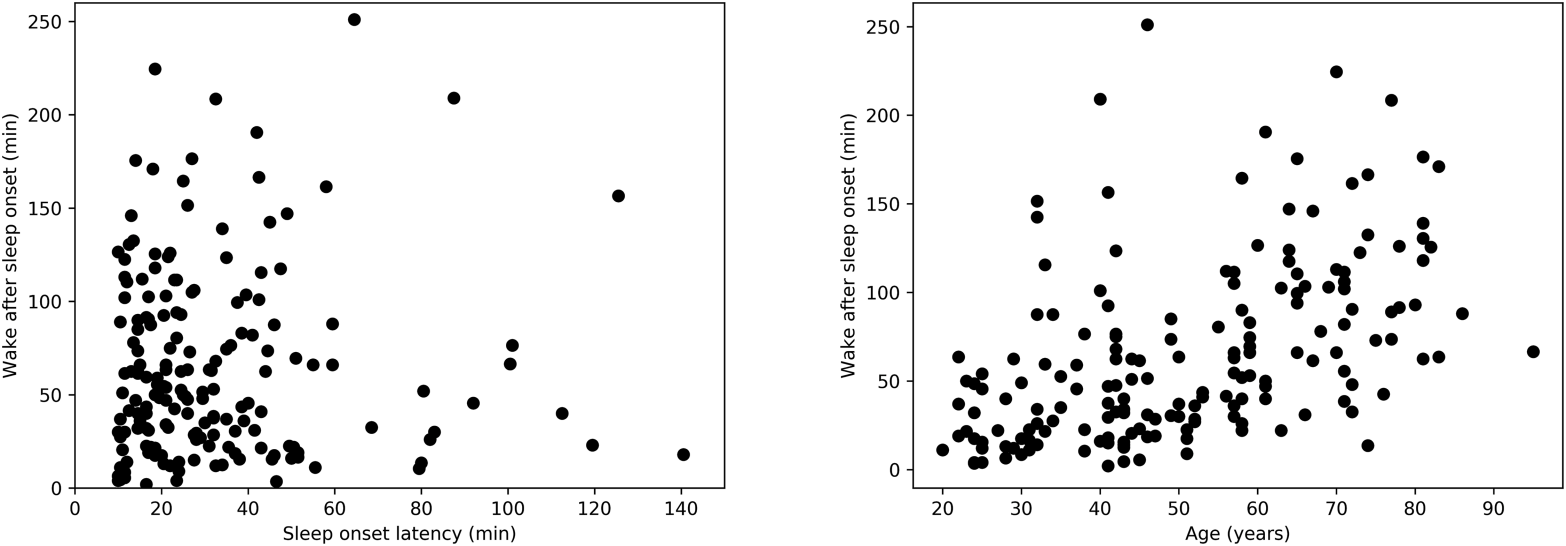
**(Left)** Relation between wake after sleep onset duration (WASO) and sleep onset latency (SOL) as measured by Spearman's correlation, *r* = −0.001, *p* = 0.97. **(Right)** Relation between WASO and age as measured by Spearman's correlation, *r* = 0.55, *p* < 0.0001.

Covariates such as age have a known effect on HRV characteristics. Techniques such as analysis of covariance (ANCOVA) are often used to account for these covariates when comparing the characteristics of different groups. However, in the case of our study, not all the assumptions of ANCOVA were met. For instance, and as explained above, age was found to be significantly different between the independent variable (WASO groups) and was further found to be positively correlated with the actual WASO (Spearman's correlation *r* = 0.55, *p* < 0.0001, Figure 2, right). Furthermore, the normality assumptions in ANCOVA were not satisfied.

Instead of ANCOVA, generalized linear models (GLM) were used. The mean value of each HRV feature in the first 10 min in bed intending to sleep, 10 min before and after sleep onset between the short and long SOL groups was compared using a GLM with gamma distribution and inverse link function [25] (experimentally found to best fit the distribution and characteristics of our data).

GLM with gamma distribution and inverse link function was also used to assess the differences in the mean values of each HRV after sleep onset between the two WASO groups while controlling for the effect of age [25]. Statistical significance was established for *p* < 0.05. Probability of superiority (PS) was used to compute effect size. For this statistic the expected value is, under the null hypothesis, that there is no difference between the groups, PS = 0.5. No correction for multiple testing, such as Bonferroni, was applied, since there is a known dependency of the HRV features on each other.

We tested separately the interactions between group and BMI and between group and sex, and we found no interactions in the SOL analysis. However, for WASO, interactions were present between group and BMI and between group and sex. Therefore, the parameters sex and BMI were included in the WASO model. Additionally, we found that age and BMI were positively correlated (Pearson's correlation *r* = 0.38, *p* < 0.001) but found no significant relation between age and sex.

For a better understanding and to visualize the results of our analysis we categorized the participants in the following age groups: 20–30, 30–40, 50–60, 60–70, 70–80, and 80–100 years, BMI groups: 16–18.5 (underweight), 18.5–25 (healthy weight), 25–30 (overweight), and 30–40 (obese) kg/m^2^, sex groups: female and male.

## 3. Results

Descriptive statistics regarding the sample size and sleep characteristics such as SOL and WASO are presented in Table 1. Table 1 also includes details regarding awake duration before sleep onset, as well as sleep duration and number of awakenings after sleep onset. We used a Mann-Whitney U-test for all three variables and we found no significant differences in these parameters between the short and long SOL groups. This supports the notion that group differences are not simply due to, for example, a different number of awakenings. Furthermore, the relation between WASO and SOL in Figure 2, left suggests that sleep onset and maintenance are separate constructs.

**Table 1:** Descriptive results for the short and long SOL groups.

	**Short SOL group**	**Long SOL group**
Sample size	107 (54 F)	69 (44 F)
age (mean ± sd) (years)	53 ± 17	48 ± 17
SOL (mean ± sd) (min)	19 ± 6	53 ± 25
WASO (mean ± sd) (min)	64 ± 46	66 ± 56
awake bSO (median [25%, 75%] (min)	10 [9.5, 10]	10 [9, 10]
asleep aSO (median [ 25%, 75%]) (min)	9.5 [8, 10]	10 [8.5, 10]
number of awakenings aSO (median [ 25%, 75%]) (min)	1 [1, 1.5]	0 [0, 1]

*aSO, 10 min after sleep onset; bSO, 10 min before sleep onset; F, Female; SOL, sleep onset latency; sd, standard deviation; WASO, wake after sleep onset*.

### 3.1. HRV Measures in Relation to Sleep Onset Latency

**Figure 3:**
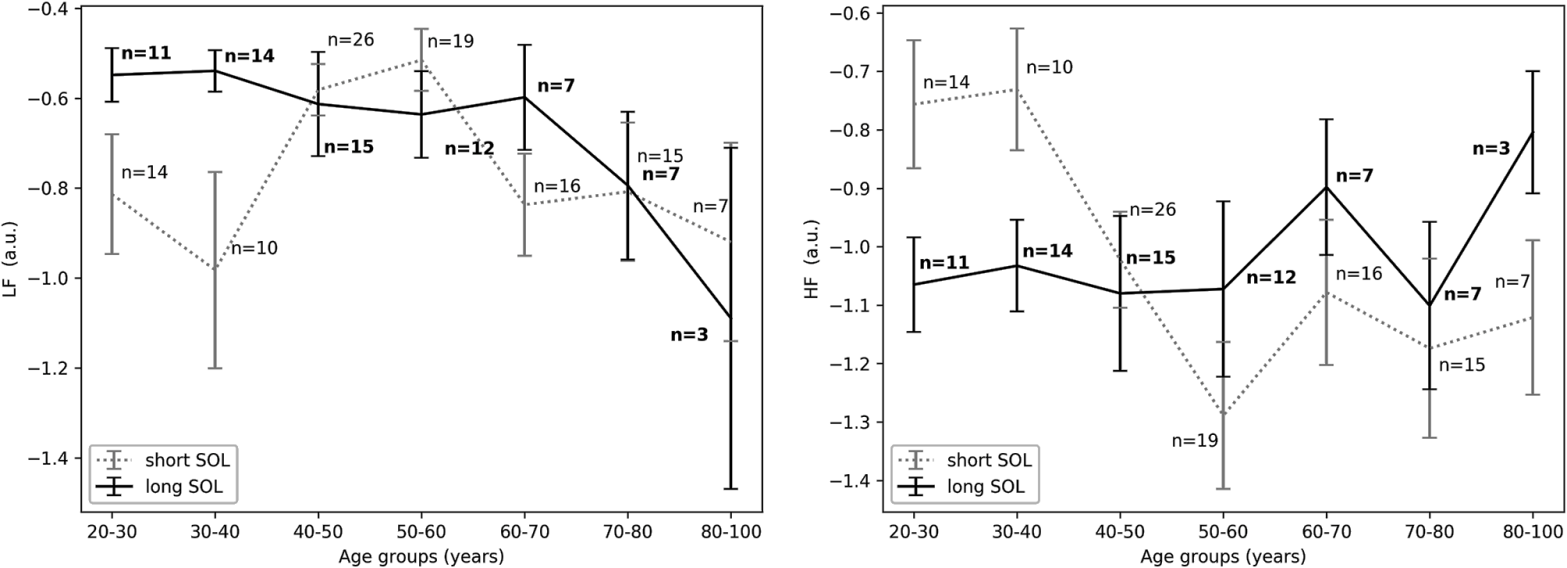
**(Left)** *LFnu* in the first 10 min in bed intending to sleep between short and long SOL for all age groups as measured by GLM model, χ^2^ = 3.71, *p* = 0.05. **(Right)** *HFnu* in the first 10 min in bed intending to sleep between short and long SOL for all age groups as measured by GLM model, χ^2^ = 5.46, *p* < 0.05. n, number of participants in the SOL group for the specific age group (bold for the long SOL group). a.u., arbitrary unit.

**Figure 4:**
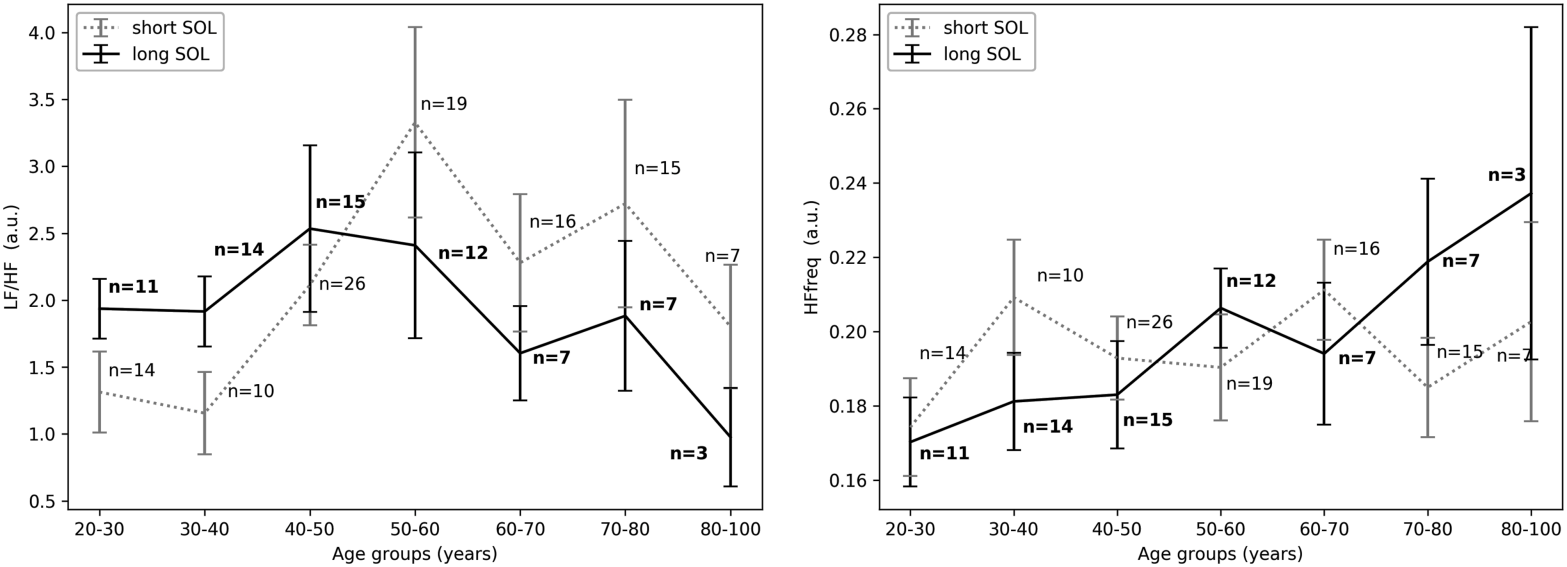
**(Left)** *LF*/*HF* in the first 10 min in bed intending to sleep between short and long SOL for all age groups as measured by GLM model, χ^2^ = 1.93, *p* = 0.17. **(Right)** *HFfreq* in the first 10 min in bed intending to sleep between short and long SOL for all age groups as measured by GLM model, χ^2^ = 3.3, *p* = 0.07. n, number of participants in the SOL group for the specific age group (bold for the long SOL group). a.u., arbitrary unit.

Results pertaining to differences in groups with long and short SOL are presented in Table 2. HRV analysis showed a significant difference in *HF*_*nu*_ in the first 10 min in bed intending to sleep as indicated by the group main effect (χ^2^ = 5.46, *p* < 0.05). The *LF*_*nu*_ and *HF*_*nu*_ values for both groups across ages are shown in Figure 3. *LF*/*HF* and *HFfreq* values in all age groups are shown in Figure 4.

**Table 2:** Results of the GLM model applied to the mean values of each HRV feature during the three time periods of interest between the short and long SOL group.

**HRV feature**	**Main effect**	***P*-value**	**PS**
*L*_*F*_*nu*_*wits*_	group: χ^2^ = 3.71	**p* = 0.05*	0.54
	age: χ^2^ = 0.02	**p* = 0.89*	
	age x group: χ^2^ = 2.5	**p* = 0.11*	
*H*_*F*_*nu*_*wits*_	group: χ^2^ = 5.46	**p* < 0.05*	0.48
	age: χ^2^ = 10.36	**p* < 0.005*	
	age x group: χ^2^ = 5.66	**p* < 0.05*	
*LF*/*HF*_*wits*_	group: χ^2^ = 1.93	**p* = 0.17*	0.52
	age: χ^2^ = 4.2	**p* < 0.05*	
	age x group: χ^2^ = 2.43	**p* = 0.12*	
*HFfreq*_*wits*_	group: χ^2^ = 3.3	**p* = 0.07*	0.49
	age: χ^2^ = 0.17	**p* = 0.68*	
	age x group: χ^2^ = 3.75	**p* = 0.05*	
*L*_*F*_*nu*_*bSO*_	group: χ^2^ = 2.76	**p* = 0.09*	0.52
	age: χ^2^ = 0.01	**p* = 0.91*	
	age x group: χ^2^ = 3.24	**p* = 0.07*	
*H*_*F*_*nu*_*bSO*_	group: χ^2^ = 3.88	**p* < 0.05*	0.49
	age: χ^2^ = 5.64	**p* < 0.05*	
	age x group: χ^2^ = 4.12	**p* < 0.05*	
*LF*/*HF*_*bSO*_	group: χ^2^ = 1.94	**p* = 0.16*	0.51
	age: χ^2^ = 2.39	**p* = 0.12*	
	age x group: χ^2^ = 2.20	**p* = 0.14*	
*HFfreq*_*bSO*_	group: χ^2^ = 2.57	**p* = 0.11*	0.51
	age: χ^2^ = 0.27	**p* = 0.60*	
	age x group: χ^2^ = 3.93	**p* < 0.05*	
*L*_*F*_*nu*_*aSO*_	group: χ^2^ = 3.88	**p* < 0.05*	0.54
	age: χ^2^ = 0.68	**p* = 0.41*	
	age x group: χ^2^ = 4.21	**p* < 0.05*	
*H*_*F*_*nu*_*aSO*_	group: χ^2^ = 4.10	**p* < 0.05*	0.47
	age: χ^2^ = 8.27	**p* < 0.005*	
	age x group: χ^2^ = 4.23	**p* < 0.05*	
*LF*/*HF*_*aSO*_	group: χ^2^ = 2.03	**p* = 0.15*	0.52
	age: χ^2^ = 2.92	**p* = 0.09*	
	age x group: χ^2^ = 2.37	**p* = 0.12*	
*HFfreq*_*aSO*_	group: χ^2^ = 3.23	**p* = 0.07*	0.47
	age: χ^2^ = 0.02	**p* = 0.87*	
	age x group: χ^2^ = 2.91	**p* = 0.09*	

*aSO, 10 min after sleep onset; bSO, 10 min before sleep onset; GLM, generalized linear model; HRV, heart rate variability; wits, the first 10 min in bed with intention to sleep; PS, probability of superiority; SOL, sleep onset latency*.

**Figure 5:**
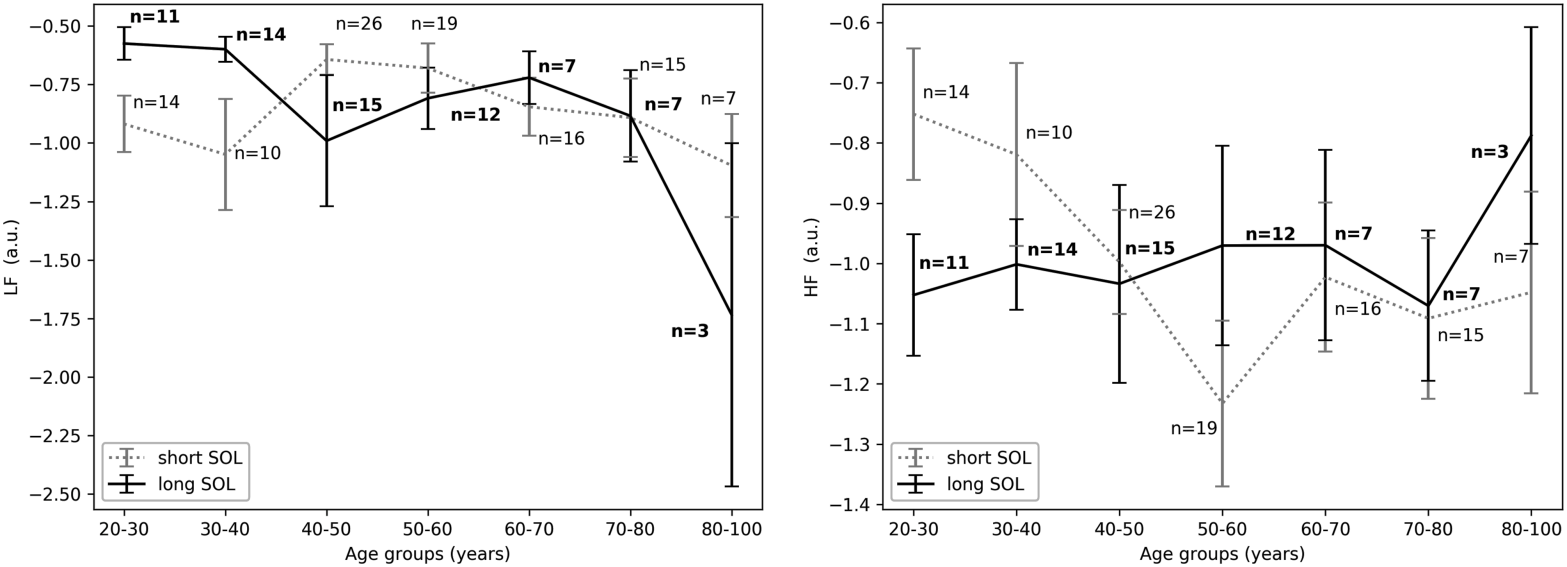
**(Left)** *LFnu* 10 min before sleep onset between short and long SOL for all age groups as measured by GLM model, χ^2^ = 2.76, *p* = 0.09. **(Right)** *HFnu* 10 min before sleep onset between short and long SOL for all age groups as measured by GLM model, χ^2^ = 3.88, *p* < 0.05. n, number of participants in the SOL group for the specific age group (bold for the long SOL group). a.u., arbitrary unit.

**Figure 6:**
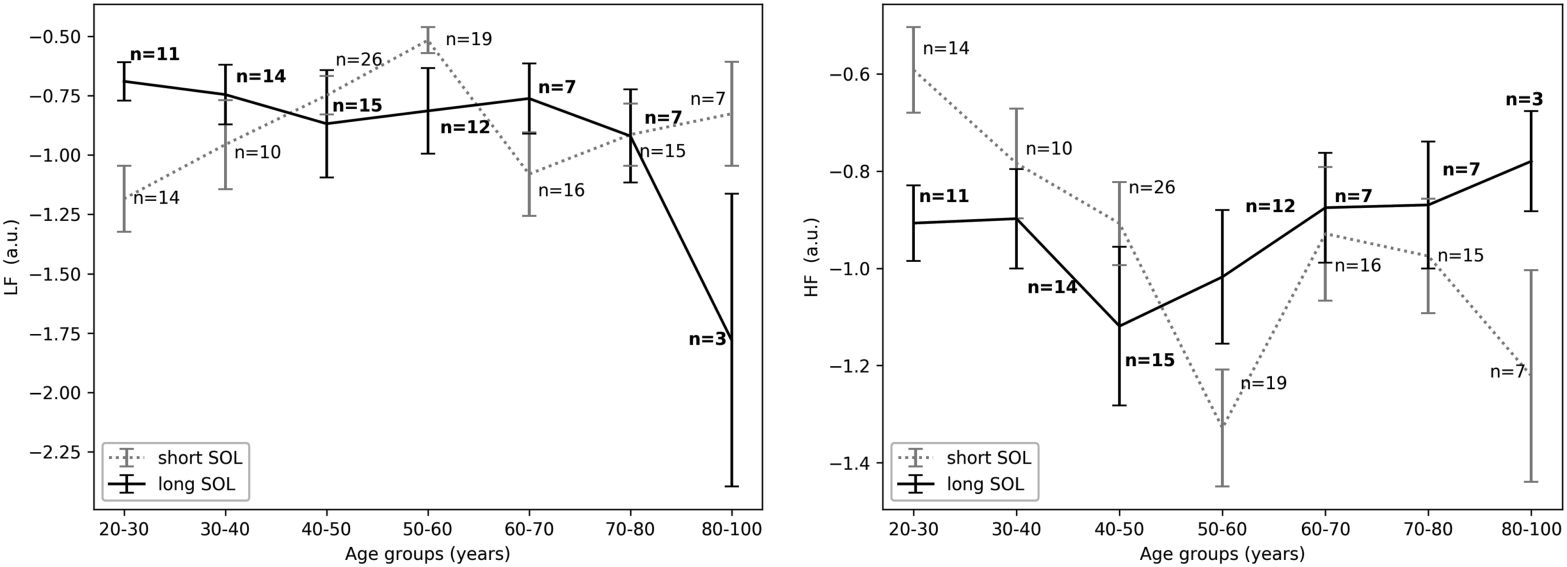
**(Left)** *LFnu* 10 min after sleep onset between short and long SOL for all age groups as measured by GLM model, χ^2^ = 3.88, *p* < 0.05. **(Right)** *HFnu* 10 min after sleep onset between short and long SOL for all age groups as measured by GLM model, χ^2^ = 4.10, *p* < 0.05. n, number of participants in the SOL group for the specific age group (bold for the long SOL group). a.u., arbitrary unit.

For the young adults (20–40 years), in the period before sleep onset, *HF*_*nu*_ was significantly higher in the short SOL group, as indicated by the group main effect (χ^2^ = 3.88, *p* < 0.05, see Figure 5, right). After sleep onset, *LF*_*nu*_ was significantly higher in the young participants (20–40 years) with a long SOL (group main effect χ^2^ = 3.88, *p* < 0.05, see Figure 6, left). *HF*_*nu*_ was lower in the long SOL group compared to the short SOL group after sleep onset (group main effect χ^2^ = 4.10, *p* < 0.05, see Figure 6, right) for the adults in age group of 20–50 years.

In all three time periods *LF*/*HF* and *HFfreq* showed no significant differences between groups. There was an interaction (group x age) effect and an age main effect for *HF*. Overall (in most time periods) there was no main effect of age and no interaction (group x age) effect for *LF* and *LF*/*HF* and *HFfreq*. Details regarding a main effect of age, as well as an interaction with age and group are presented in Table 2. Model coefficients and standard errors are presented in Table S1.

### 3.2. HRV Measures in Relation to Wake After Sleep Onset

**Figure 7:**
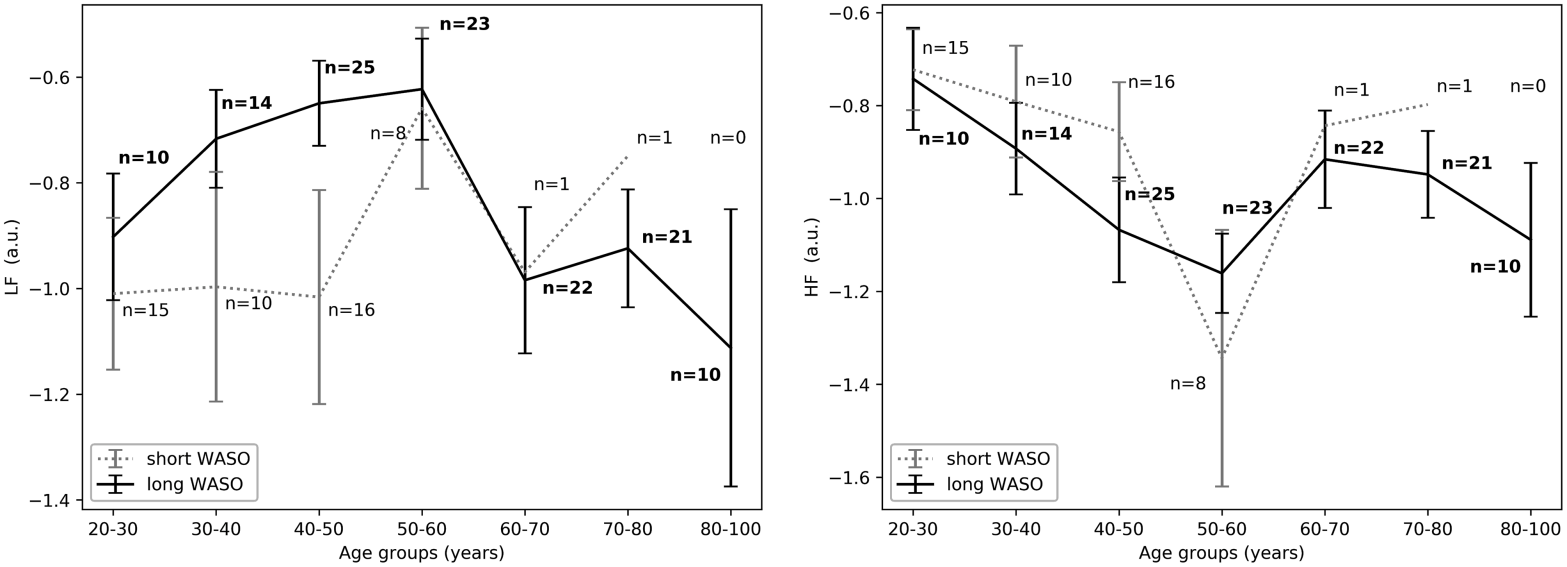
**(Left)** *LF*_*nu*_ 10 min after sleep onset between short and long WASO groups for a cutoff of 30 min for all age groups as measured by GLM model, χ^2^ = 3.40, *p* = 0.07. **(Right)** *HF*_*nu*_ 10 min after sleep onset between short and long WASO groups for a cutoff of 30 min for all age groups as measured by GLM model, χ^2^ = 4.37, *p* < 0.05. n, number of participants in the SOL group for the specific age group (bold for the long WASO group). a.u., arbitrary unit.

Descriptive statistics including overall sleep characteristics are shown in Table 3, together with details regarding sleep duration and number of awakenings after sleep onset. The latter two variables were not significantly different between groups with long and short WASO. Consistent with the literature, we observed a positive relation between WASO and age (Spearman's correlation *r* = 0.55, *p* < 0.0001; Figure 2, right). HRV analysis showed a significant difference in *HF*_*nu*_ after sleep onset between short and long WASO groups (cutoff between groups of 30 min; group main effect χ^2^ = 4.37, *p* < 0.05). *LF*/*HF* was also significantly different (group main effect: χ^2^ = 4.30, *p* < 0.05), while *HF*_*nu*_ and *HFfreq* showed no significant differences (see Table S2). There was a main effect of sex for *HF* and *LF*/*HF* and a main effect of BMI for *LF*/*HF*. Details regarding all features including model coefficients and standard errors are presented in Table S2. Figure 7 shows *LF*_*nu*_ and *HF*_*nu*_ after sleep onset for all age groups. In the long WASO group, *LF*_*nu*_ increased with age up to 60 years, while after 60 years it decreased with age (see Figure 4, left). Figure 7, right shows a decrease of *HF*_*nu*_ with age up to 60 years in both groups.

**Table 3:** Descriptive results for the short and long WASO groups.

	**Short WASO group**	**Long WASO group**
Sample size	51 (33 F)	125 (65 F)
age (mean ± sd) (years)	39 ± 12	56 ± 17
SOL (mean ± sd) (min)	36 ± 28	31 ± 21
WASO (mean ± sd) (min)	17 8	85 46
asleep aSO (median [25%, 75%]) (min)	10 [9, 10]	9.5 [7.5, 10]
number of awakenings aSO (median [25%, 75%]) (min)	0 [0, 1]	1 [0, 1]

*aSO, 10 min after sleep onset; F, Female; SOL, sleep onset latency; sd, standard deviation; WASO, wake after sleep onset*.

**Figure 8:**
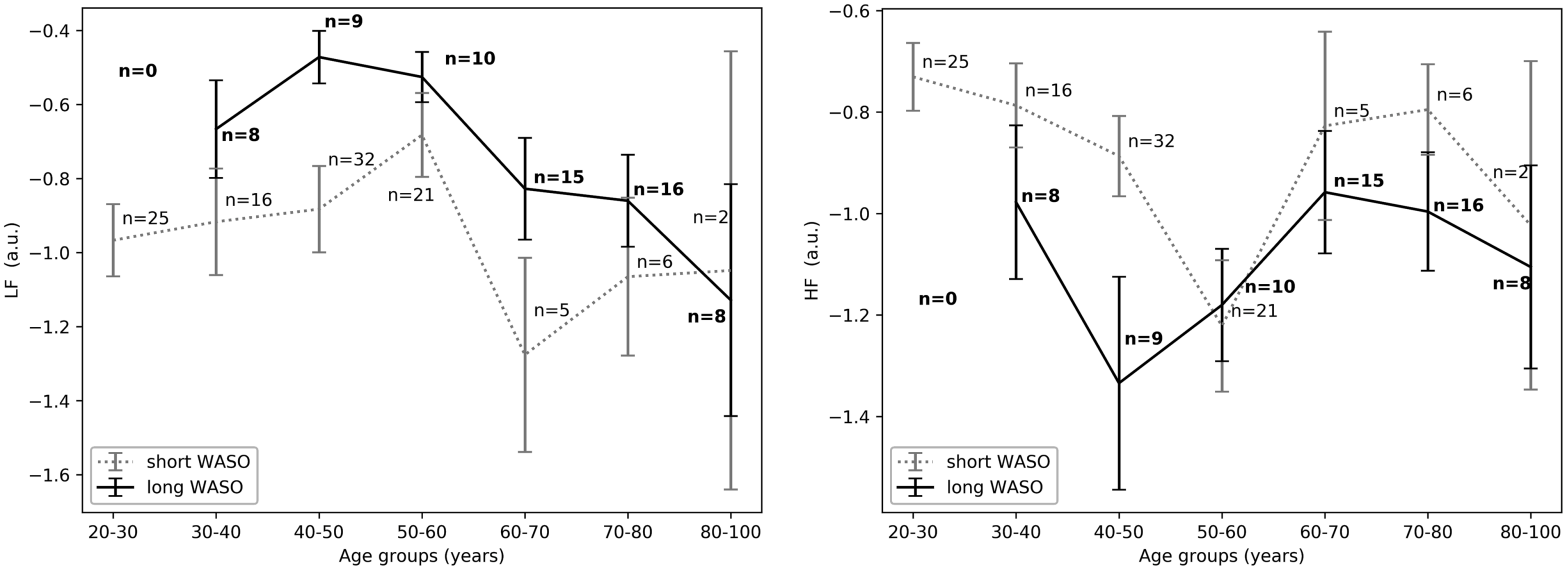
**(Left)** *LF*_*nu*_ 10 min after sleep onset between the short and the long WASO group for a cutoff of 66 min for all age groups as measured by GLM model, χ^2^ = 11.92, *p* < 0.001. **(Right)** *HF*_*nu*_ 10 min after sleep onset between the short and the long WASO group for a cutoff of 66 min for all age groups as measured by GLM model, χ^2^ = 8.27, *p* < 0.005. n, number of participants in the WASO group for the specific age group (bold for the long WASO group). a.u., arbitrary unit.

**Figure 9:**
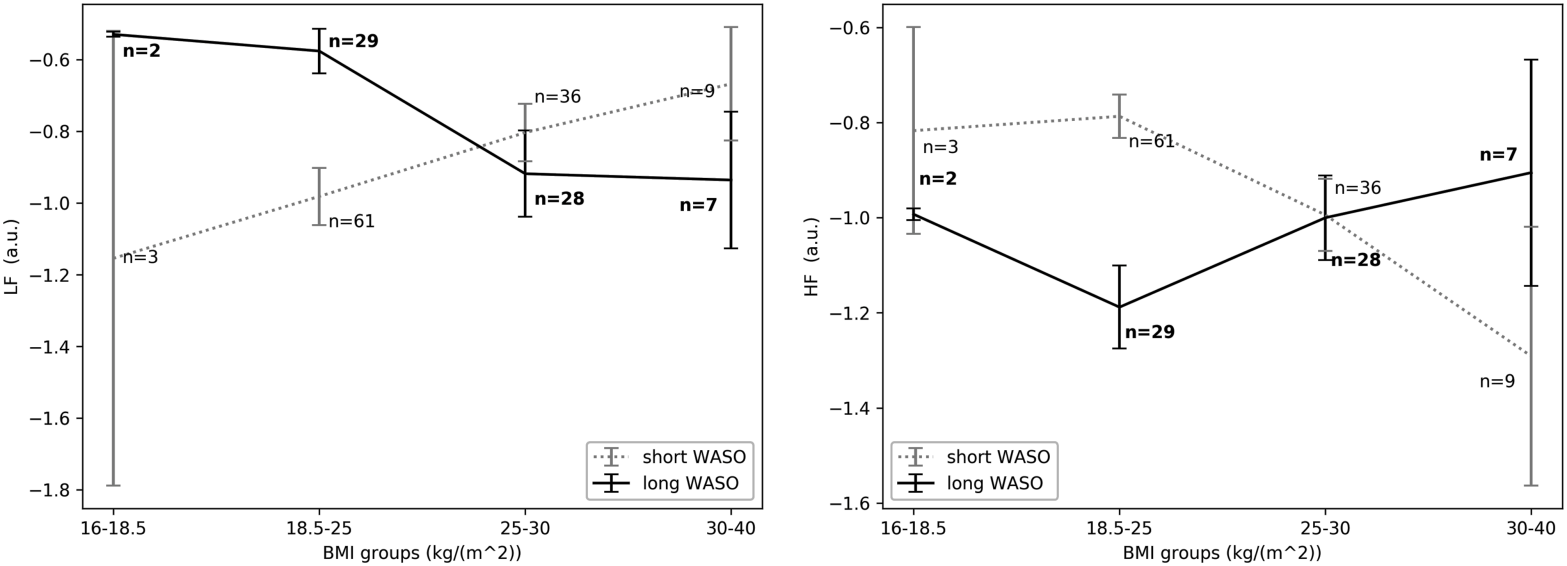
**(Left)** *LF*_*nu*_ 10 min after sleep onset between the short and the long WASO group for a cutoff of 66 min for all BMI groups as measured by GLM model, χ^2^ = 11.92, *p* < 0.001. **(Right)** *HF*_*nu*_ 10 min after sleep onset between the short and the long WASO group for a cutoff of 66 min for all BMI groups as measured by GLM model, χ^2^ = 8.27, *p* < 0.005. n, number of participants in the WASO group for the specific age group (bold for the long WASO group). a.u., arbitrary unit.

**Figure 10:**
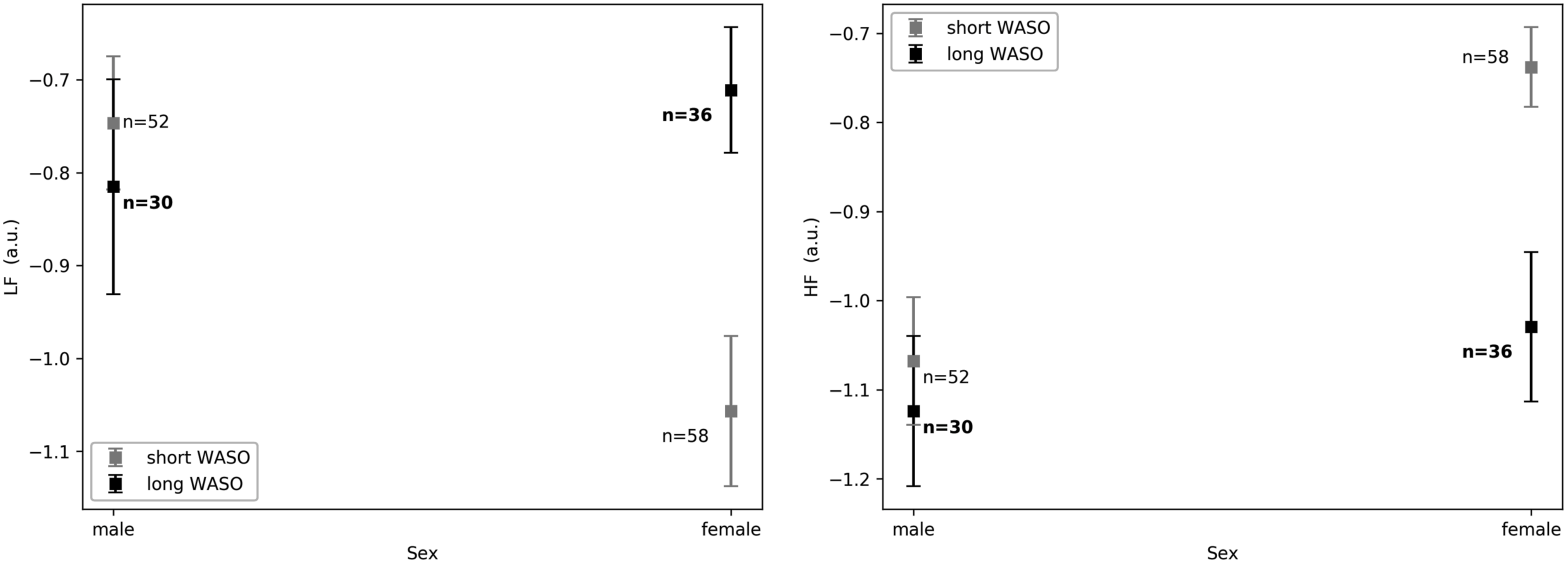
**(Left)** *LF*_*nu*_ 10 min after sleep onset between the short and the long WASO group for a cutoff of 66 min for female and men as measured by GLM model, χ^2^ = 11.92, *p* < 0.001. **(Right)** *HF*_*nu*_ 10 min after sleep onset between the short and the long WASO group for a cutoff of 66 min for female and male as measured by GLM model, χ^2^ = 8.27, *p* < 0.005. n, number of participants in the WASO group for the specific age group (bold for the long WASO group). a.u., arbitrary unit.

The exploration of different cutoffs for defining short and long WASO showed that *LF*_*nu*_, *HF*_*nu*_, and *LF*/*HF* differed between the long and the short WASO groups for the cutoffs of 41.5, 53, and 66 min (see Table S6). The highest effect size and group main effect for *LF*_*nu*_, *HF*_*nu*_, and *LF*/*HF* was seen with a WASO cutoff of 66 min (see Tables S3, S4, S6). All details regarding *LF*_*nu*_, *HF*_*nu*_, *HFfreq*, and *LF*/*HF* features including model coefficients and standard errors are presented in Supplementary material (see Tables S3–S6). Figure 8, left shows that *LF*_*nu*_ was significantly higher in the long WASO group as compared to the short WASO over all age groups, with a WASO cutoff of 66 min. The same behavior (except for the 50–60 age group) was observed for *LF*/*HF*. Accordingly, Figure 8, right shows that *HF*_*nu*_ was lower in the long WASO group as compared to the short WASO in almost all age groups. There was no main effect of sex in the WASO groups in *LF*_*nu*_ for all cutoffs, while there was an effect for *HF*_*nu*_ and *LF*/*HF* in the WASO cutoffs of 41.5, 53, and 66 min. A main effect of BMI was observed only for *LF*/*HF* and there was an interaction effect of group x BMI for *LF*_*nu*_, *HF*_*nu*_, and *LF*/*HF*. Interaction (group x sex) and (group x BMI) effects for features *LF*_*nu*_, *LF*/*HF*, and *HF*_*nu*_ in the WASO cutoffs of 53 and 66 min were observed. Details are presented in Tables S3–S6. Figure 9 shows *LF*_*nu*_ and *HF*_*nu*_ features for different BMI groups. We observe a higher *LF*_*nu*_ and a lower *HF*_*nu*_ in the long WASO group for the underweight and healthy weight BMI groups. For the overweight group HRV differences are not significant. Figure 10 shows *LF*_*nu*_ and *HF*_*nu*_ features between female and men. A higher *LF*_*nu*_ and a lower *HF*_*nu*_ in the long WASO group is seen in female group but not in the male participants.

## 4. Discussion

A combination of physiological, cognitive and behavioral changes happen during the transition from wake to sleep (Nano et al., 2018). In this work, we addressed the ANS changes as expressed by the spectral power of HRV. In accordance with the literature, *HF*_*nu*_ reflects the respiration-driven modulation of sinus rhythm, and has been commonly used as an index of tonic vagal drive (1996; Nano et al., 2017; Shaffer and Ginsberg, 2017). *HFfreq* has been shown to serve as an index of respiratory frequency (Thayer et al., 2002). *LF*/*HF* appears to provide an estimate of sympathovagal balance (1996; Dodds et al., 2017; Nano et al., 2017; Shaffer and Ginsberg, 2017). *LF*_*nu*_ has been used as a marker of sympathetic modulation, especially when expressed in normalized units (1996; Nano et al., 2017; Shaffer and Ginsberg, 2017). Researchers have also advocated that *LF*_*nu*_ reflects both sympathetic and parasympathetic activity (Burr, 2007; Dodds et al., 2017). However, the physiological significance of the lower frequency bands such as *LFnu* and *LF*/*HF* is still debated and caution is required when interpreting these measures (Burr, 2007; Billman, 2013; Heathers and Goodwin, 2017). Therefore, even though HRV measures are increasingly being used as a marker of cardiac autonomic activity during sleep, we are aware that HRV analysis has limitations especially regarding interpretation. Several recent studies highlight those limitations and support that normalized spectral measures reflect more an ANS balance measure than isolated sympathetic and parasympathetic indices (summarized in Burr, 2007; Heathers and Goodwin, 2017).

Our results suggest that participants with difficulties initiating sleep (long SOL) are more aroused in the first 10 min they intend to sleep, before sleep onset, and after sleep onset when compared to participants in the short SOL group (*HF*_*nu*_ significantly lower in participants with long SOL). This hypothesis seems to be supported by the fact that *LF*_*nu*_ was significantly higher in the long SOL group after sleep onset as compared to the short SOL group. The interaction with age and age main effect observed for *HF* as well as the interaction plots of HRV features with age (Figures 3–5) indicate that our hypothesis of an increased state of arousal in the long SOL group was mainly confirmed in the younger age groups. This was supported by a increased *LF*_*nu*_ and a decreased *HF*_*nu*_.

In all three examined time periods there was an effect of age in *HF*_*nu*_ but not in *LF*_*nu*_. These results are in agreement with the literature as it is known that there is a significant reduction in the expression of parasympathetic activity in the HRV characteristics with age, more specifically in the *HF*_*nu*_ band (Stratton et al., 2003). In Figure 3 we observe that in the short SOL group, *HF*_*nu*_ decreases with age up to 60 years, while after 60 years there is a slight increase after which it flattens out. The decrease of heart rate with age has been confirmed by several studies in the literature (O'Brien et al., 1986; Larson et al., 2013; Porta et al., 2014). Similar findings are reported in a recent study by Kuo et al. (2016). The authors (Kuo et al., 2016) investigated changes and interactions of autonomic functioning with age and sleep architecture and found that ANS has different effects on sleep architecture before and after 50 years of age. More specifically, a negative correlation of *HF*_*nu*_ with age up to the age of 50 year was present, while this phenomenon was not found among the 50–79 year age group; as a consequence, a positive relation with age was present but not significant. As seen in Figure 3, right, this trend it is not present in the long SOL group and a lower variability of *HF*_*nu*_ with age is observed. This could be related to the state of arousal mentioned earlier.

Our hypothesis of an increased state of arousal in participants with long SOL compared to short SOL cannot be fully confirmed by all features in all time periods. We realize that HRV features have limitations with regard to interpretation, as firstly they remain surrogates or estimators of autonomic activity and secondly due to the complicated interactions between sympathetic and parasympathetic nerve activity. However, overall our results (e.g., based on *HFnu*) are compatible with an increased state of arousal, especially for the young adults (20–40 years).

For the WASO cutoff of 30 min often used in the literature, we found a significant difference in *HF*_*nu*_ and *LF*/*HF* but not in *LF*_*nu*_ and *HFfreq* between the two groups. It is worth mentioning that this comparison was mainly done in subjects below 60 years of age, as only 2 subjects above 60 had a WASO ≤ 30 min (Figure 7). When considering different cutoffs for defining short and long WASO, results showed that *LF*_*nu*_, *HF*_*nu*_, and *LF*/*HF* differed between the long and the short WASO groups for the cutoffs of 41.5, 53, and 66 min, all longer than the 30 min often used. The highest effect size and group main effect for all three features (*LF*_*nu*_, *HF*_*nu*_, and *LF*/*HF*) was achieved for a WASO cutoff of 66 min.

Overall there was no main effect of age for all features. For the 66 min cutoff we noticed that the minimum difference for both *HF*_*nu*_ and *LF*_*nu*_ was observed in the age group of 50–60 years (Figure 8). One explanation could be a relation with meno- and andropause (Brockbank et al., 2000; Schwarz et al., 2011). It is known that sleep quality and duration may decrease with age. Indeed, in our analysis older people had a higher WASO. It is of course possible that long WASO in the elderly may be influenced by other age-related factors besides autonomic arousal, such as deteriorated circadian physiology and perhaps reduced sleep need, even though the latter is still under debate (Klerman et al., 2008; Mander et al., 2017).

Our results suggest that age, BMI as well as sex have an influence in the expression of HRV features in relation to WASO, but to a different degree, depending on the HRV feature. The exact contribution is difficult to ascertain in this data set because of correlations between the factors. It is difficult to assess such high level interactions in our data set, as the set is not balanced and matched with respect to age, sex, and BMI.

*HFfreq* overall did not show any significant difference with respect to short and long SOL and WASO with the exception of the 66 min WASO cutoff. *HFfreq* has been shown to serve as a surrogate of respiratory frequency. It is known that *HFfreq* varies from subject to subject and it is mainly driven by respiratory sinus arrhythmia (RSA). RSA decreases with age (Stratton et al., 2003) due to the weakening cardiorespiratory coupling on older people. So it is likely that the accuracy of *HFfreq* as an estimator of respiration frequency decreases. Furthermore, no normalization was applied in this feature and since it varies from person to person, this suggests that differences in estimated respiratory rates reflect mostly the differences between participants rather than the effect of the ANS has on that value.

To the best of our knowledge, there is no available literature investigating HRV between normal sleepers with long and short SOL or WASO. We assessed HRV as an indicator of autonomic functioning in normal sleepers in relation to differences in sleep onset latency and wake after sleep onset, as a “model” for pathological problems with sleep onset or maintenance, i.e., insomnia. So far, studies on autonomic function in insomnia disorder have not yielded a consistent picture. Recent reviews discuss studies comparing insomnia vs. normal sleep, showing inconsistent results (Dodds et al., 2017; Nano et al., 2017). While Dodds et al. suggest that impairments in HRV cannot be confirmed in insomnia patients, we argued in our overview that there are overall differences in cardiovascular activity between insomniacs and controls, as long as patients were grouped according to phenotype, for instance with respect to objective sleep duration. For example, Miller et al. found attenuated parasympathetic activity at sleep onset in insomniacs with short sleep duration compared to those with normal total sleep duration (Miller et al., 2016). However, this behavior was not present when insomnia with short sleep duration was further divided into type-A (defined by high WASO) and type-B (defined by high SOL and medium WASO). These uncertainties prompted us to assess HRV dynamics in participants without diagnosed insomnia but with varying degree of SOL and/or WASO. In addition, the effect of the first night recording in the sleep lab can be considered a stressor possibly explaining the state of arousal observed both the long SOL as well as long WASO group. It is also possible that (some of) our participants might even show early signs of sleep disturbances without qualifying for insomnia diagnosis. In any case, we show that a stressor can be reflected in the HRV autonomic activity which could highlight the mechanisms of long SOL or WASO possibly more clearly than in more severe insomnia patients where some autonomic characteristics could be masked by several other factors. Future studies in larger cohorts including diagnosed insomnia of variable severity may shed light on the generalizability of our findings and the associated mechanisms underlying long SOL and WASO.

Limitations of this study include the fact that we investigated only one definition of sleep onset. It would be interesting to compare different definitions of sleep onset in future studies. This should be done in separate, extended datasets. In that case, analysis could be extended to include more HRV features [such as VLF, RR variance, non-linear HRV indices (Richman and Moorman, 2000; Maestri et al., 2007)]. Finally, the exploration of pathological sleep including formally diagnosed insomnia would be insightful to explore if there is an association with the chronic aspect of the disorder. We included a large dataset with a wide variety of ages. However, generalizability of our findings need to be explored in future studies. For instance, it would be interesting to explore a continuous analysis of SOL and/or WASO with a large and matched dataset with respect to age, sex, and BMI in order to explore the higher level interactions between these three parameters. Furthermore, our analysis yield interesting new hypotheses underlying mechanisms for insomnia that should be explored in future studies. In addition, subjective indicators of sleep disturbances should be included in future studies as it is known that cardiac arousal may contribute to subjective complaints (Stepanski., 2002; Halász et al., 2004; Silvani et al., 2015), while abnormal PSG-based SOL or WASO might not always be observed (Baglioni et al., 2014; Wei et al., 2017).

In summary, this study supports the hypothesis that participants with difficulties in maintaining sleep are more hyperaroused in the beginning of sleep compared to those without difficulties, as supported by the elevated *LF*_*nu*_ and decreased *HF*_*nu*_ regardless of the age of the participant. These results had a higher effect size and were more distinctive using a cutoff of 66 min as compared to using a cutoff of 30 min. The results also support the hypothesis of a state of arousal for participants with problems initiating sleep (long SOL), especially for young adults (20–40 years), in the first 10 min in bed intending to sleep, and just before and after sleep onset as compared to participants in the short SOL group. Based on our findings we can argue that autonomic cardiac activity has a predictive value with respect to sleep characteristics pertaining to sleep onset and maintenance.

## Data Availability Statement

The Siesta dataset analyzed in this study was obtained from a third party and is described in the study of Klösch et al. (2001). Information regarding the licenses/restrictions and access to this dataset should be directed to the Siesta group (contact: https://www.thesiestagroup.com/contact.php). The remaining datasets used for this manuscript are not publicly available because it was not foreseen in the informed consent from all human participants involved in the study. Requests to access the datasets should be directed to Pedro Fonseca (pedro.fonseca@philips.com).

## Ethics Statement

The studies involving human participants were reviewed and approved by the Internal Committee of Biomedical Experiments of Philips Research. The participants provided their written informed consent to participate in this study.

## Author Contributions

MN performed the investigation, formal and statistical analysis, wrote the first draft of the manuscript, and did project administration. PF, SO, RV, and RA supervised this study. All authors contributed to the conception and design of the study, methodology, manuscript revision, read and approved the submitted version.

### Conflict of Interest

MN, PF, and RA declare to be affiliated with Philips Research. RV reports ownership of shares in Nemo Healthcare, outside the submitted work. The remaining author declares that the research was conducted in the absence of any commercial or financial relationships that could be construed as a potential conflict of interest.
