# Supplementary material for: Lying Awake at Night: Cardiac Autonomic Activity in Relation to Sleep Onset and Maintenance
Source: Front Neurosci. 2020 Jan 15;13:1405. doi: 10.3389/fnins.2019.01405 (PMC6974549; doi:10.3389/fnins.2019.01405)
Supplement: Supplementary file 1 [file Data_Sheet_1.pdf]

Supplementary Material:

Lying Awake at Night: Cardiac Autonomic Activity in Relation to Sleep Onset and Maintenance

1 SUPPLEMENTARY TABLES

Table S1. Results of the GLM model applied to the mean values of each HRV feature during the three time periods of interest between the short and long SOL group

Cutoffs	Main effect	P-value	Model coefficient	Standard error	PS
LF_{nuwits}	group: χ^2 = 3.71	$p = 0.05$	-0.85	0.44	0.54
	age: χ^2 = 0.02	$p = 0.89$	-0.001	0.01	
	age x group: χ^2 = 2.5	$p = 0.11$	0.01	0.01	
HF_{nuwits}	group: χ^2 = 5.46	$p <$ ***0.05***	0.47	0.20	0.48
	age: χ^2 = 10.36	$p <$ ***0.005***	0.01	0.002	
	age x group: χ^2 = 5.66	$p <$ ***0.05***	-0.01	0.004	
LF/HF_{wits}	group: χ^2 = 1.93	$p = 0.17$	-0.29	0.21	0.52
	age: χ^2 = 4.2	$p <$ ***0.05***	-0.01	0.002	
	age x group: χ^2 = 2.43	$p = 0.12$	0.01	0.004	
$HFfreq_{wits}$	group: χ^2 = 3.3	$p = 0.07$	1.24	0.68	0.49
	age: χ^2 = 0.17	$p = 0.68$	-0.003	0.01	
	age x group: χ^2 = 3.75	$p = 0.05$	-0.02	0.01	
LF_{nubSO}	group: χ^2 = 2.76	$p = 0.09$	-0.70	0.42	0.52
	age: χ^2 = 0.01	$p = 0.91$	0.001	0.01	
	age x group: χ^2 = 3.24	$p = 0.07$	0.01	0.01	
HF_{nubSO}	group: χ^2 = 3.88	$p <$ ***0.05***	0.45	0.22	0.49
	age: χ^2 = 5.64	$p <$ ***0.05***	0.01	0.003	
	age x group: χ^2 = 4.12	$p <$ ***0.05***	-0.01	0.004	
LF/HF_{bSO}	group: χ^2 = 1.94	$p = 0.16$	-0.31	0.22	0.51
	age: χ^2 = 2.39	$p = 0.12$	-0.003	0.003	
	age x group: χ^2 = 2.20	$p = 0.14$	0.01	0.004	
$HFfreq_{bSO}$	group: χ^2 = 2.57	$p = 0.11$	0.91	0.56	0.51
	age: χ^2 = 0.27	$p = 0.60$	0.004	0.01	
	age x group: χ^2 = 3.93	$p <$ ***0.05***	-0.02	0.01	
LF_{nuaSO}	group: χ^2 = 3.88	$p <$ ***0.05***	-0.73	0.37	0.54
	age: χ^2 = 0.68	$p = 0.41$	-0.004	0.004	
	age x group: χ^2 = 4.21	$p <$ ***0.05***	0. 01	0.01	
HF_{nuaSO}	group: χ^2 = 4.10	$p <$ ***0.05***	0.51	0.24	0.47
	age: χ^2 = 8.27	$p <$ ***0.005***	0.01	0.003	
	age x group: χ^2 = 4.23	$p <$ ***0.05***	-0.01	0.01	
LF/HF_{aSO}	group: χ^2 = 2.03	$p = 0.15$	-0.40	0.27	0.52
	age: χ^2 = 2.92	$p = 0.09$	-0.01	0.003	
	age x group: χ^2 = 2.37	$p = 0.12$	0.01	0.01	
$HFfreq_{aSO}$	group: χ^2 = 3.23	$p = 0.07$	0.89	0.49	0.47
	age: χ^2 = 0.02	$p = 0.87$	-0.001	0.01	
	age x group: χ^2 = 2.91	$p = 0.09$	-0.02	0.01	

aSO: ten minutes after sleep onset, bSO: ten minutes before sleep onset, GLM: generalized linear model, HRV: heart rate variability, $wits$: the first 10 minutes in bed with intention to sleep, PS: probability of superiority, SOL: sleep onset latency

Table S2. Results of the GLM model applied to the mean values of each HRV feature after sleep onset between the short and long WASO group (WASO cutoff of 30 min)

Cutoffs	Main effect	P-value	Model coefficient	Standard error	PS
LF_{nuaSO}	group: χ^2 = 3.40	$p = 0.07$	-1.58	0.86	0.56
	age: χ^2 = 0.09	$p = 0.76$	0.001	-0.003	
	age x group: χ^2 = 1.18	$p = 0.28$	0.01	0.01	
	BMI: χ^2 = 1.10	$p = 0.30$	-0.03	0.03	
	group x BMI: χ^2 = 1.43	$p = 0.23$	0.04	0.04	
	sex: χ^2 = 2.64	$p = 0.10$	0.36	0.22	
	group x sex: χ^2 = 1.27	$p = 0.26$	-0.30	0.27	
HF_{nuaSO}	group: χ^2 = 4.37	$p <$ ***0.05***	1.19	0.57	0.43
	age: χ^2 = 1.00	$p = 0.32$	0.01	0.01	
	age x group: χ^2 = 0.26	$p = 0.61$	-0.04	0.01	
	BMI: χ^2 = 3.11	$p = 0.08$	0.03	0.02	
	group x BMI: χ^2 = 3.31	$p = 0.07$	-0.04	0.02	
	sex: χ^2 = 6.51	$p <$ ***0.05***	- 0.40	0.16	
	group x sex: χ^2 = 1.37	$p = 0.24$	0.21	0.18	
LF/HF_{aSO}	group: χ^2 = 4.30	$p <$ ***0.05***	-1.19	0.57	0.56
	age: χ^2 = 1.37	$p = 0.24$	-0.01	0.01	
	age x group: χ^2 = 0.97	$p = 0.33$	0.01	0.01	
	BMI: χ^2 = 7.31	$p < 0.01$	-0.03	0.01	
	group x BMI: χ^2 = 3.6	$p = 0.06$	0.04	0.02	
	sex: χ^2 = 5.06	$p <$ ***0.05***	0.42	0.19	
	group x sex: χ^2 = 2.01	$p = 0.16$	-0.30	0.21	
$HFfreq_{aSO}$	group: χ^2 = 0.29	$p = 0.59$	-0.65	1.20	0.57
	age: χ^2 = 0.12	$p = 0.73$	0.01	0.02	
	age x group: χ^2 = 0.21	$p = 0.65$	0.01	0.01	
	BMI: χ^2 = 2.25	$p = 0.13$	-0.07	0.04	
	group x BMI: χ^2 = 0.27	$p = 0.60$	0.03	0.05	
	sex: χ^2 = 1.30	$p = 0.25$	-0.37	0.32	
	group x sex: χ^2 = 0.15	$p = 0.70$	0.14	0.37	

aSO: ten minutes after sleep onset, GLM: generalized linear model, HRV: heart rate variability, PS: probability of superiority, WASO: wake after sleep onset duration

Table S3. Results for LF_{nu} after sleep onset for different WASO cutoffs

Cutoffs	Main effect	P-value	Model coefficient	Standard error	PS
25th %ile (28.5 min)	group: χ^2 = 3.45	$p = 0.06$	-1.67	0.89	0.55
	age: χ^2 = 0.10	$p = 0.75$	-0.002	0.01	
	age x group: χ^2 = 1.19	$p = 0.27$	0.01	0.01	
	BMI: χ^2 = 1.14	$p = 0.29$	-0.03	0.03	
	group x BMI: χ^2 = 1.56	$p = 0.21$	0.05	0.04	
	sex: χ^2 = 2.47	$p = 0.12$	0.36	0.23	
	group x sex: χ^2 = 1.15	$p = 0.28$	-0.29	0.27	
40th %ile (41.5 min)	group: χ^2 = 4.34	$p <$ ***0.05***	-1.76	0.84	0.57
	age: χ^2 = 0.08	$p = 0.78$	-0.002	0.01	
	age x group: χ^2 = 2.34	$p = 0.13$	0.01	0.01	
	BMI: χ^2 = 1.05	$p = 0.31$	-0.03	0.03	
	group x BMI: χ^2 = 1.37	$p = 0.24$	0.04	0.03	
	sex: χ^2 = 2.09	$p = 0.15$	0.26	0.18	
	group x sex: χ^2 = 0.71	$p = 0.40$	-0.20	0.24	
50th %ile (53 min)	group: χ^2 = 9.90	$p <$ ***0.005***	-2.63	0.83	0.56
	age: χ^2 = 0.02	$p = 0.88$	-0.001	0.01	
	age x group: χ^2 = 3.51	$p = 0.06$	0.01	0.01	
	BMI: χ^2 = 1.96	$p = 0.16$	-0.03	0.02	
	group x BMI: χ^2 = 4.55	$p <$ ***0.05***	0.07	0.03	
	sex: χ^2 = 3.04	$p = 0.08$	0.28	0.16	
	group x sex: χ^2 = 1.63	$p = 0.20$	-0.30	0.27	
60th %ile (66 min)	group: χ^2 = 11.92	$p <$ ***0.001***	-2.99	0.87	0.59
	age: χ^2 = 0.19	$p = 0.66$	0.002	0.004	
	age x group: χ^2 = 3.46	$p = 0.06$	0.02	0.01	
	BMI: χ^2 = 1.96	$p = 0.16$	-0.03	0.02	
	group x BMI: χ^2 = 5.91	$p <$ ***0.05***	0.08	0.03	
	sex: χ^2 = 5.60	$p <$ ***0.05***	0.34	0.14	
	group x sex: χ^2 = 5.07	$p <$ ***0.05***	-0.53	0.24	
75th %ile (98 min)	group: χ^2 = 3.15	$p = 0.08$	-2.03	1.15	0.58
	age: χ^2 = 0.73	$p = 0.39$	0.003	0.004	
	age x group: χ^2 = 1.13	$p = 0.29$	0.01	0.01	
	BMI: χ^2 = 0.40	$p = 0.53$	-0.01	0.02	
	group x BMI: χ^2 = 0.94	$p = 0.33$	0.04	0.04	
	sex: χ^2 = 3.33	$p = 0.07$	0.25	0.13	
	group x sex: χ^2 = 0.99	$p = 0.32$	-0.31	0.31	

PS: probability of superiority, WASO: wake after sleep onset duration

Table S4. Results for LF/HF after sleep onset for different WASO cutoffs

Cutoffs	Main effect	P-value	Model coefficient	Standard error	PS
25th %ile (28.5 min)	group: χ^2 = 4.66	$p <$ ***0.05***	-1.28	0.59	0.54
	age: χ^2 = 1.66	$p = 0.20$	-0.01	0.01	
	age x group: χ^2 = 1.11	$p = 0.29$	0.01	0.01	
	BMI: χ^2 = 7.41	$p <$ ***0.01***	-0.03	0.01	
	group x BMI: χ^2 = 4.02	$p = 0.05$	0.04	0.02	
	sex: χ^2 = 3.22	$p = 0.07$	0.36	0.20	
	group x sex: χ^2 = 098	$p = 0.32$	-0.22	0.22	
40th %ile (41.5 min)	group: χ^2 = 6.46	$p <$ ***0.05***	-1.29	0.51	0.58
	age: χ^2 = 1.53	$p = 0.22$	-0.01	0.01	
	age x group: χ^2 = 1.35	$p = 0.25$	0.01	0.01	
	BMI: χ^2 = 11.51	$p <$ ***0.001***	-0.03	0.01	
	group x BMI: χ^2 = 5.01	$p <$ ***0.05***	0.04	0.02	
	sex: χ^2 = 8.05	$p <$ ***0.005***	0.45	0.16	
	group x sex: χ^2 = 4.16	$p <$ ***0.05***	-0.37	0.18	
50th %ile (53 min)	group: χ^2 = 8.61	$p <$ ***0.005***	-1.38	0.47	0.58
	age: χ^2 = 2.42	$p = 0.12$	-0.01	0.004	
	age x group: χ^2 = 2.42	$p = 0.12$	0.01	0.01	
	BMI: χ^2 = 11.59	$p <$ ***0.001***	-0.03	0.01	
	group x BMI: χ^2 = 5.62	$p <$ ***0.05***	0.04	0.02	
	sex: χ^2 = 10.52	$p <$ ***0.01***	0.44	0.13	
	group x sex: χ^2 = 5.83	$p <$ ***0.05***	-0.39	0.16	
60th %ile (66 min)	group: χ^2 = 10.82	$p <$ ***0.001***	-1.50	0.46	0.60
	age: χ^2 =01.94	$p = 0.16$	- 0.004	0.003	
	age x group: χ^2 = 3.17	$p = 0.08$	0.01	0.004	
	BMI: χ^2 = 13.11	$p <$ ***0.001***	-0.03	0.01	
	group x BMI: χ^2 = 5.51	$p <$ ***0.05***	0.04	0.02	
	sex: χ^2 = 11.10	$p <$ ***0.001***	0.39	0.12	
	group x sex: χ^2 = 5.34	$p <$ ***0.05***	-0.35	0.15	
75th %ile (98 min)	group: χ^2 = 1.30	$p = 0.25$	-0.71	0.62	0.60
	age: χ^2 = 0.39	$p = 0.54$	-0.001	0.003	
	age x group: χ^2 = 0.78	$p = 0.38$	0.01	0.01	
	BMI: χ^2 = 3.44	$p = 0.06$	-0.02	0.01	
	group x BMI: χ^2 = 0.33	$p = 0.57$	0.01	0.03	
	sex: χ^2 = 5.81	$p <$ ***0.05***	0.27	0.11	
	group x sex: χ^2 = 1.18	$p = 0.28$	-0.20	0.17	

PS: probability of superiority, WASO: wake after sleep onset duration

Table S5. Results for $HFfreq$ after sleep onset for different WASO cutoffs

Cutoffs	Main effect	P-value	Model coefficient	Standard error	PS
25th %ile (28.5 min)	group: χ^2 = 0.04	$p = 0.83$	-0.27	1.27	0.58
	age: χ^2 = 0.09	$p = 0.77$	0.004	0.02	
	age x group: χ^2 = 0.14	$p = 0.71$	-0.01	0.02	
	BMI: χ^2 = 1.31	$p = 0.25$	-0.05	0.05	
	group x BMI: χ^2 = 0.04	$p = 0.85$	0.01	0.05	
	sex: χ^2 = 0.69	$p = 0.41$	-0.28	0.34	
	group x sex: χ^2 = 0.01	$p = 0.91$	0.04	0.39	
40th %ile (41.5 min)	group: χ^2 = 0.65	$p = 0.42$	0.90	1.12	0.48
	age: χ^2 = 3.09	$p = 0.08$	-0.02	0.01	
	age x group: χ^2 = 1.40	$p = 0.24$	0.01	0.01	
	BMI: χ^2 = 0.10	$p = 0.75$	-0.01	0.03	
	group x BMI: χ^2 = 1.27	$p = 0.26$	-0.05	0.05	
	sex: χ^2 = 0.79	$p = 0.37$	-0.22	0.25	
	group x sex: χ^2 = 0.001	$p = 0.98$	-0.01	0.32	
50th %ile (53 min)	group: χ^2 = 1.28	$p = 0.26$	1.29	1.14	0.48
	age: χ^2 = 0.90	$p = 0.34$	-0.01	0.01	
	age x group: χ^2 = 0.001	$p = 0.98$	-0.0003	0.01	
	BMI: χ^2 = 0.52	$p = 0.47$	-0.02	0.03	
	group x BMI: χ^2 = 0.88	$p = 0.35$	-0.04	0.05	
	sex: χ^2 = 1.78	$p = 0.18$	-0.30	0.23	
	group x sex: χ^2 = 0.35	$p = 0.55$	0.19	0.32	
60th %ile (66 min)	group: χ^2 = 4.55	$p <$ ***0.05***	2.49	1.17	0.47
	age: χ^2 = 0.66	$p = 0.42$	-0.01	0.01	
	age x group: χ^2 = 0.31	$p = 0.58$	-0.01	0.01	
	BMI: χ^2 = 0.19	$p = 0.66$	-0.01	0.03	
	group x BMI: χ^2 = 2.72	$p = 0.10$	-0.08	0.05	
	sex: χ^2 = 2.17	$p = 0.14$	-0.30	0.20	
	group x sex: χ^2 = 0.60	$p = 0.44$	0.25	0.33	
75th %ile (98 min)	group: χ^2 = 1.37	$p = 0.24$	1.55	1.33	0.50
	age: χ^2 = 0.30	$p = 0.58$	-0.003	0.01	
	age x group: χ^2 = 0.70	$p = 0.40$	-0.01	0.01	
	BMI: χ^2 = 1.49	$p = 0.22$	-0.03	0.03	
	group x BMI: χ^2 = 0.31	$p = 0.58$	-0.03	0.05	
	sex: χ^2 = 1.97	$p = 0.16$	-0.26	0.19	
	group x sex: χ^2 = 0.13	$p = 0.72$	0.14	0.38	

PS: probability of superiority, WASO: wake after sleep onset duration

Table S6. Results for HF_{nu} after sleep onset for different WASO cutoffs

Cutoffs	Main effect	P-value	Model coefficient	Standard error	PS
25th %ile (28.5 min)	group: χ^2 = 5.12	$p <$ ***0.05***	1.32	0.58	0.44
	age: χ^2 = 1.43	$p = 0.23$	0.01	0.01	
	age x group: χ^2 = 0.43	$p = 0.27$	-0.005	0.01	
	BMI: χ^2 = 3.44	$p = 0.06$	0.03	0.02	
	group x BMI: χ^2 = 3.85	$p = 0.05$	-0.04	0.02	
	sex: χ^2 = 4.27	$p <$ ***0.05***	-0.35	0.17	
	group x sex: χ^2 = 0.56	$p = 0.46$	0.14	0.19	
40th %ile (41.5 min)	group: χ^2 = 5.67	$p <$ ***0.05***	1.28	0.54	0.42
	age: χ^2 = 3.51	$p = 0.06$	0.01	0.01	
	age x group: χ^2 = 1.83	$p = 0.18$	-0.01	0.01	
	BMI: χ^2 = 3.16	$p = 0.08$	0.03	0.02	
	group x BMI: χ^2 = 3.24	$p = 0.07$	-0.04	0.02	
	sex: χ^2 = 10.73	$p <$ ***0.005***	-0.43	0.13	
	group x sex: χ^2 = 3.52	$p = 0.06$	0.30	0.16	
50th %ile (53 min)	group: χ^2 = 6.83	$p <$ ***0.01***	1.38	0.53	0.42
	age: χ^2 = 4.64	$p <$ ***0.05***	0.01	0.004	
	age x group: χ^2 = 2.55	$p = 0.11$	-0.01	0.01	
	BMI: χ^2 = 2.89	$p = 0.09$	0.03	0.02	
	group x BMI: χ^2 = 3.70	$p <$ ***0.05***	-0.04	0.02	
	sex: χ^2 = 12.90	$p <$ ***0.001***	-0.42	0.12	
	group x sex: χ^2 = 4.45	$p <$ ***0.05***	0.32	0.15	
60th %ile (66 min)	group: χ^2 = 8.27	$p <$ ***0.005***	1.50	0.52	0.38
	age: χ^2 = 3.64	$p = 0.06$	0.01	0.003	
	age x group: χ^2 = 2.70	$p = 0.10$	-0.01	0.01	
	BMI: χ^2 = 3.29	$p = 0.07$	0.03	0.01	
	group x BMI: χ^2 = 3.71	$p <$ ***0.05***	-0.04	0.02	
	sex: χ^2 = 13.57	$p <$ ***0.001***	-0.38	0.10	
	group x sex: χ^2 = 3.81	$p = 0.05$	0.29	0.15	
75th %ile (98 min)	group: χ^2 = 1.62	$p = 0.20$	0.76	0.59	0.39
	age: χ^2 = 2.85	$p = 0.09$	0.005	0.003	
	age x group: χ^2 = 1.34	$p = 0.25$	-0.01	0.01	
	BMI: χ^2 = 0.77	$p = 0.38$	0.01	0.01	
	group x BMI: χ^2 = 0.29	$p = 0.59$	-0.01	0.02	
	sex: χ^2 = 2.51	$p = 0.09$	-0.28	0.09	
	group x sex: χ^2 = 1.63	$p = 0.20$	0.21	0.17	

PS: probability of superiority, WASO: wake after sleep onset duration
